# Supplementary material for: Comparison of Polymerase Chain Reaction and Urine Culture in the Evaluation of Patients with Complex Urinary Tract Infections
Source: Biology (Basel). 2024 Apr 13;13(4):257. doi: 10.3390/biology13040257 (PMC11048588; doi:10.3390/biology13040257)
Supplement: Supplementary file 1 [file biology-13-00257-s001.zip › biology-2933815-supplementary.pdf]

Comparison of Polymerase Chain Reaction to Urine Culture in the Evaluation of Patients with Complex Urinary Tract Infections

Supplement A – Protocol for Specimen Collection and Processing

Deepak A. Kapoor, Mara R. Holton, Jason Hafron, Rima Aljundi, Bernadette Zwaans, Mitchell Hollander

Table of Contents

Protocol for clean-catch mid-stream urine specimen collection....2

Purpose....2

Policy....2

Supplies....2

Procedure....2

Protocol for urine collection via straight or indwelling catheter....3

Purpose....3

Policy....3

Supplies....3

Procedure....3

Additional Details Regarding Urine Culture and PCR Protocols....4

Protocol for clean-catch mid-stream urine specimen collection

PURPOSE

- To provide clinical personnel with guidelines and patient instructions on how to properly collect a Clean-Catch Mid-stream Urine Specimen.
- This method flushes the normal flora from the urethra and urinary meatus before the specimen is collected which eliminates the need to collect the specimen by catheterization.

POLICY

- All patients must be instructed by the MA on how to collect a Clean-Catch Mid-stream Specimen every time they are required to provide a urine sample.
- Handling of specimens should be performed according to defined procedures. The proper collection and handling of a urine specimen is important for obtaining accurate results.
- Urine specimens which will not be tested within two hours need to be refrigerated to avoid bacterial overgrowth.
- All staff must wear gloves when handling urine specimens.
- Specimen cups must be labeled (not on the lid) with two patient identifiers. (Initials and medical record number)

SUPPLIES

- Sterile specimen container ;
- Label with patient's initials and medical record number;
- 1 towelette for males, 3 for females.

PROCEDURE

- Provide the patient with a labeled, sterile urine specimen cup and the appropriate number of towelettes (1 for males, 3 for females).
- The patient should be instructed where to leave the specimen.
- Explain the process for a clean-catch midstream collection and inform the patient that written instructions are posted in the restroom for their reference. (see Figure 1 below)
- Ensure that all patient questions are answered.

MICHIGAN INSTITUTE OF UROLOGY

Patient Instructions: How to Collect a Clean-Catch Urine

***Wash hands in warm soapy water before you begin**

Female	Male
1. Open specimen container; do not touch the inside of the cup or the cover.<br>2. Open towelette. Separate the folds of the urinary opening with fingers and clean inside using one towelette, moving from the front to the back.<br>3. Clean one side and discard towelette.<br>4. New towelette: Clean middle and discard towelette.<br>5. New towelette: Clean other side and discard towelette.<br>6. Continue to hold the folds open and begin urinating into the toilet.<br>7. Bring the specimen container into the stream and collect a clean, **mid-stream** specimen.<br>8. Finish urination into the toilet.<br>9. Secure the cover.	1. Open specimen container; do not touch the inside of the cup or the cover.<br>2. Open towelette. Retract foreskin (if present) and use the towelette to clean the entire head of the penis.<br>3. Begin urinating into the toilet and bring the container into the stream to collect a clean, **mid-stream** specimen.<br>4. Finish urination into the toilet.<br>5. Secure the cover.

Figure 1: Patient instruction sheet posted at site of collection

Supplement A: Protocol for Specimen Collection and Processing

Protocol for urine collection via straight or indwelling catheter[1]

PURPOSE

- To provide clinical personnel with guidelines on how to properly collect an adequate amount of urine (15-20cc) when using a straight or indwelling catheter for urinalysis or culture.

POLICY

- The physician/provider is responsible for giving the order for a urinalysis and or urine culture. If the patient is unable to give a clean catch specimen, an order must be given to catheterize the patient and collect the specimen. This can be accomplished by:
    - Straight catheterization via red rubber catheter; or
    - By temporarily clamping an indwelling catheter if present.

SUPPLIES

- Straight Catheter
- 3 OB Towel / Alcohol pad
- Gauze 4x4's
- Lubricant
- Blue pad/ White drape
- Sterile Specimen cup
- PPE--gloves, eye protection
- Catheter plug/clamp (if warranted)

PROCEDURE

Urine collection via straight (red rubber) catheter

General Instructions

- PPE must be worn during any manipulation of a catheter.
- Catheter insertion is completed using "clean technique."
- Identify the patient using patient name and date of birth.
- Ensure that the patient is not allergic to latex, iodine, or betadine.
    - If the patient is allergic or sensitive to latex use a latex free catheter.
- Explain the procedure to the patient; maintain patient's privacy and dignity.
- Before insertion:
    - Dispense the lubricating gel on to the gauze 4x4's.
    - Open OB Towelette.
    - Open the catheter package using sterile technique.
    - Don protective eye wear
    - Place "blue pad" underneath patient to collect any fluid drainage.

Female insertion

- Position female patient into a frog-leg pose.
- Separate the labia using the non-dominant hand and visualize the meatus.
- Take one OB towel and wipe on side of the labia from top to bottom and discard, repeat on the opposite side and discard, wipe down the middle using a third OB towel and discard.
- Using the dominant hand, cover the tip of the catheter with lubricant.
- Insert the catheter approximately three inches into the urethra, wait for urine to flow into the sterile container and collect approximately 15-20cc of urine.
- Avoid contact between the catheter and the sterile container.
- Withdraw the catheter slowly and discard into a clear bag.
    - If grossly contaminated it must be discarded in a biohazard bag.
- Using a preprinted label, label the specimen container before leaving the room.

---

[1] Although included in the protocol summary for specimen collection via catheterization for completeness, in our practice specimens obtained via indwelling catheter are not sent for analysis via polymerase chain reaction and no specimens included in this data were obtained via indwelling catheter.

Male insertion

- Position the male patient into a supine pose.
- Retract the foreskin, if present, and hold the shaft of the penis with the non-dominant hand.
- Take one OB towel, using a circular motion; wipe the glans from the meatus outward. Discard. Repeat with 2 more OB towels and discard.
- Grasp the penis in an upright position and insert the lubricated catheter firmly into the meatus, advancing the catheter into the urethra to the bifurcation at the "Y" of the catheter.
- If resistance is met when advancing the catheter do not attempt forceful insertion, contact the physician or provider for further instruction.
- Wait for urine to flow into the sterile container and collect approximately 15-20cc of urine.
- Avoid contact between the catheter and the sterile container.
- Withdraw the catheter slowly and discard into a clear bag.
    - If grossly contaminated it must be discarded in a biohazard bag.
- If the foreskin was retracted, reposition it once catheter is removed.
- Using a preprinted label, label container before leaving the room.

Collecting a urine sample from an indwelling catheter

- Disconnect foley catheter from collecting system.
- Plug the foley catheter for 10-15 minutes.
    - Monitor the patient to ensure he/she is not experiencing discomfort.
- Obtain sterile container and open lid.
- Pinch or clamp the foley catheter.
- Remove catheter plug/clamp.
- Clean opening of catheter with alcohol and allow to dry.
- Obtain adequate amount of urine (15-20cc), preventing touching sterile container, close lid tightly.
- Using preprinted label, label container before leaving the room.

Additional Details Regarding Urine Culture and PCR Protocols

The clinical microbiology lab protocol for urine culture is dependent on the urine sample type. For urine samples collected by voided clean catch, inoculation of both blood agar plate and MacConkey agar is performed with 1ul of patient urine sample. When an isolate is identified, if there is <10,000 CFU/mL, identification based on gram stain or morphology is performed. If there is >10,000 CFU/mL, definitive identification and antibiotic susceptibility is performed.

For urine samples collected by straight catheter or catheterized urine, inoculation of both blood agar plate and MacConkey agar is performed with 10 ul of patient urine sample. When a isolate is identified, if there is 100 to 1,000 CFU/mL, identification based on gram stain or morphology is performed. If there is >1,000 CFU/mL, definitive identification and antibiotic susceptibility is performed.

For the urinary tract PCR diagnostic test, DNA nucleic acid extraction was performed with 800 µL of patient urine sample using ThermoFisher's MagMax Ultra kit and enzyme mix for lysis and run on the Kingfisher Flex platform.

Specific studies to determine the correlation between DNA/copies mL of pathogen identified by PCR and CFU/mL of pathogen identified by urine culture were not performed given the variability in counting number of bacterial cells (generally represented by CFU per mL), versus number of copies of genomic DNA per mL.[2] The analytical validation of the PCR assay was performed utilizing DNA copies/mL of pathogen and antibiotic resistance gene. However, a component of the clinical validation did include PCR assay correlation with patient samples which were positive by urine culture and had identification and antibiotic susceptibility performed.

---

[2] Morpeth SC, Huggett JF, Murdoch DR, Scott JA. Making standards for quantitative real-time pneumococcal PCR. Biomol Detect Quantif. 2014 Dec 4;2:1-3.
